# Changes in Mental Health during Three Waves of the COVID-19 Pandemic in Slovakia: Neurotypical Children versus Children with Autism Spectrum Disorder and Their Parents

**DOI:** 10.3390/ijerph191911849

**Published:** 2022-09-20

**Authors:** Katarína Polónyiová, Barbara Rašková, Daniela Ostatníková

**Affiliations:** Academic Research Centre for Autism, Institute of Physiology, Faculty of Medicine, Comenius University, 813 72 Bratislava, Slovakia; barbara.raskova@fmed.uniba.sk (B.R.); daniela.ostatnikova@fmed.uniba.sk (D.O.)

**Keywords:** COVID-19, lockdown, mental health, autism spectrum disorder

## Abstract

The main goal of our research was to monitor changes in the mental health of Slovak families with children with autism spectrum disorder (ASD) compared to families with neurotypical children during three waves of the COVID-19 pandemic. We focused on the prevalence of depression, anxiety, stress and different stressors of parents. In children, we explored maladaptive behavior and the availability of interventions for children with ASD. The data were collected using an extensive questionnaire including the Depression, Anxiety, and Stress Scale-42 questionnaire (DASS-42) and two subscales of the Vineland Adaptive Behavior Scales (VABS-3). The research sample consisted of a total of 506 parents, 236 of whom have a child with ASD. Parents of children with ASD reported elevated anxiety during the first wave, while changes were found in parents of neurotypical children. During the second wave, the prevalence of anxiety, depression and stress experienced by parents in both groups increased, but significantly more in parents with ASD children. The internalizing maladaptive behavior of children with ASD also increased. During the third wave, no significant differences between the groups of parents were found in stress and anxiety, but parents of ASD children scored higher in depression. Externalized maladaptive behavior of neurotypical children increased, with minimal changes in children with ASD, which can be explained by the improved therapy availability for children with ASD, also observed in our study.

## 1. Introduction

The impact that the COVID-19 pandemic had on the mental health of people evoked substantial interest among experts as well as the public in the topic of mental health and inspired numerous research studies. The pandemic development has been dynamic and, while most countries and individuals reacted similarly at the beginning of the pandemic, the circumstances in each country began to vary over time. Among the many factors that need to be considered when generalizing the information are the severity of the epidemiologic situation and the quality of the healthcare system of a country, differences in safety measures introduced (lockdown strictness and length, school closures, etc.) and the way they are presented by the country leaders, as well as access to relevant information in general [1].

During the first wave in the spring of 2020, Slovakia, like neighboring central European countries, swiftly introduced strict safety measures, including the closure of all services, schools and shops except groceries and pharmacies. As a result, the first wave was short-lived, and Slovakia was considered one of the countries that managed it well. After all measures were lifted during the summer, an uncontrolled growth of cases in September 2020 started the second wave, which lasted until spring 2021 [2]. During the second wave, Slovakia was significantly more affected, both in terms of infection and death rates and as a burden on the whole healthcare system, schools and the economy. The third wave began in the fall of 2021. It was similar to the second in severity but its duration was shorter (until the end of December 2021), and the introduced safety measures were more relaxed, with schools opened for in-person learning for the first time since the beginning of the pandemic.

Research studies exploring how the mental health of children and students in Slovakia and neighboring countries was affected by the pandemic are mostly limited to information about university students. There was a two-fold increase in depression and anxiety among Slovak college students during the second wave of the COVID-19 pandemic compared to 2018, especially for younger students, and those showing higher stress or loneliness, and lower resilience [3]. Ochnik et al. [4] reported that college students from Poland, Slovenia, Czech Republic, Ukraine, Russia, Germany, Turkey, Israel and Colombia all generally suffered from high stress and mild anxiety and depression symptoms, with varying severity levels between the countries. Rogowska et al. [5] note that anxiety, stress, physical health and life satisfaction of college students worsened significantly across three waves of the COVID-19 pandemic in Poland, and anxiety symptoms were found predominantly in women, individuals with poor health, or those suffering from high stress.

Depressive symptoms of different severity were found in Slovak adults, especially females and younger individuals [6]. During the first wave of the pandemic, slightly reduced well-being and moderate levels of stress, anxiety and loneliness were reported in Slovak parents, with a significant decrease in well-being, anxiety and loneliness during the second wave [7]. Deterioration of mental health over time during the epidemic was also found in working adults in the Czech Republic, and the proportion of individuals experiencing at least one mental disorder was highest during the second wave [8]. More depression, anxiety and stress symptoms were found in women, people under the age of 35 [9], those with interrupted working status, younger adults or students, and those having lost a job or with only elementary education [10].

### COVID-19 Pandemic and Children with Autism Spectrum Disorder

Previous COVID-19 related research from various countries of the world already showed that the pandemic had a particular impact on the mental health of patients with prior mental health conditions [11], including autism spectrum disorder [12,13]. Autism spectrum disorder (ASD) is a pervasive neurodevelopmental disorder defined by deficits in reciprocal communication and social interaction, and restricted or repetitive behaviors and interests [14]. There are additional symptoms bringing specific challenges during the pandemic—difficulty coping with change, tolerating uncertainty and generating adaptive strategies, intellectual deficits or executive function impairments, poor emotion recognition or regulation skills and commonly co-occurring mental health conditions such as ADHD, anxiety or depression [15]. Reportedly, almost 50% of the individuals with ASD had difficulty fully comprehending the concept of the COVID-19 pandemic and necessary safety measures [16], with verbal children being paradoxically at even higher risk for related psychiatric problems after hearing large amounts of negative information with lowered ability to process it emotionally [17].

Children with ASD were more than three times as likely to experience negative consequences of the pandemic, and were mainly negatively affected by routine changes, as compared to neurotypical children who were primarily affected by social isolation [18]. Sudden disruption of the daily routines, often crucial to individuals with ASD, provoked anxiety among many children with ASD [19]. Furthermore, constant and unpredictable changes in safety measures, general uncertainty of the situation, and seeing some people not following the rules, which autistic individuals tend to perceive as “black and white”, left individuals with ASD confused [15].

Pandemic-related school closures and suddenly switching to online education also brought different challenges to children with ASD. Children with ASD lost access to all interventions and specialized education previously provided by the schools, though on the other hand, the amount of necessary interpersonal interactions required of them also decreased. Thus, the closure of schools resulted in reduced stress in some autistic individuals and increased stress in others [20]. In general, higher levels of aggression, anxiety, irritability, hyperactivity, impulsivity and lack of attention were found in individuals with ASD when compared to neurotypical children, and higher levels of repetitive, restrictive and stereotyped behaviors were also found in the participants with ASD during the lockdown period when compared to the time prior to the pandemic [21]. The increased presence of restrictive, repetitive, self-injurious, compulsive and ritualistic behaviors can be understood as an alternative way of coping with disrupted routines [22]. Within two months of the onset of COVID-19, almost 60% of children with ASD experienced psychiatric problems, over 50% of children developed new symptoms and about 45% had a worsening of their pre-existing psychiatric disorder [17].

Not all children with ASD were affected to the same degree. The factors increasing their vulnerability to psychological distress include higher age, the severity of the disorder, the amount of support needed and the disruption of prior support during the pandemic [23], intellectual disability, specific learning disorder or communication disorder [24], level of understanding of COVID-19, COVID-19 illness in the family, low family income, and elevated parental depression and anxiety symptoms [17]. Levels of stress, anxiety, depression, PTSD-related symptoms and tiredness were also higher during the second pandemic wave, when compared to the first one [25].

Parenting children with ASD is also demanding and even before the pandemic, families of children with ASD experienced more stress than families of neurotypical children or even children with cerebral palsy or Down syndrome [26]. During the COVID-19 pandemic, studies reported increased stress in caregivers of children with ASD, with the highest stressors being problems with emotion regulation of their child, coping with stress, loss of structure and established routines [27,28]. Severity of the core ASD symptoms children exhibited during the pandemic were a good predictor of their quality of life as well as the quality of life of their parents [29]. Compared to parents of neurotypical children, more parents of children with ASD exhibited symptoms of anxiety and depression, related to generally having lower levels of resilience and positive coping and using negative coping strategies more often [30]. Concerns about loss of institutional support were especially frequent in parents of children having co-occurring intellectual disability and in need of specialized education [28].

The main goal of the current study is to analyze changes in the mental health of Slovak families across three waves of the COVID-19 pandemic, with the main focus on comparing families with neurotypical children and children with ASD.

## 2. Materials and Methods

### 2.1. Measures and Instruments

A robust parent survey was created, containing three main sections: demographic questions, a section about the mental health of parents, and one about that of their children. To measure the prevalence of depression, anxiety and stress symptoms of parents and caregivers, we used Depression Anxiety and Stress Scale DASS-42 [31]. DASS-42 is a self-report questionnaire composed of 42 statements that participants rate on a scale of 0–3, from “0 = Did not apply to me at all” to “3 = Applied to me very much, or most of the time”. Depression, Anxiety, and Stress scales are each calculated by the sum of 14 corresponding items. Higher scores indicate higher symptoms severity. Furthermore, we asked parents what were the most prominent stressors they experienced during each wave of the pandemic, choosing from a list inspired by [32]: lockdown duration, fears of the infection, frustration and boredom, unavailability of goods and services, insufficient or unclear information, work insecurity/finances and more demanding child care.

In the section about their children, we used two subscales of the Vineland questionnaire [33] measuring the prevalence of internalizing (such as distress, sadness, apathy or social withdrawal) and externalizing (attention deficits, problems with self-regulation, aggression or disobedience) maladaptive behavior. Vineland Adaptive Behavior Scales—Third Edition is a questionnaire used to evaluate the adaptive functioning of a child with its three broad domains: communication, daily living skills, and socialization. The internalizing and externalizing maladaptive behavior subscales consist of 13 and 11 items, respectively. The items are rated on a scale from 0–2 meaning the described behavior “0 = Does not occur at all”, “1 = Sometimes” or “2 = Often”. The final score for each scale is the sum of all items. Additionally, we asked parents of children with ASD which therapeutic interventions and in what form (online/in-person) was their child receiving.

### 2.2. Study Design

The study design was in accordance with the Ethics Committee of the Faculty of Medicine, Comenius University in Bratislava. Participation in the research was anonymous, voluntary, unpaid and the participants entered after agreeing with the informed consent form at the beginning of our questionnaire. The survey was first sent to parents of children diagnosed in the Academic Research Centre for Autism at the Institute of Physiology of the Faculty of Medicine, Comenius University in Bratislava a year prior, and to parents of neurotypical children willing to partake. During the following pandemic waves, participants from previous data collections who agreed to be contacted again were surveyed first and, afterwards, the questionnaire was shared forward.

### 2.3. Research Sample

In three consecutive cross-sectional surveys, we gathered answers from 506 parents and caregivers, 236 of whom have children with ASD. A total of 179 participants were surveyed during the first wave, 153 during the second wave and 174 during the third wave of COVID-19 pandemic in Slovakia. Further demographic information about our sample can be found in the Table 1.

### 2.4. Data Collection

The first data collection took place from June to July of 2020, asking participants to evaluate their experience in the time period from March and June 2020 which is considered a first wave of the COVID-19 pandemic in Slovakia. The second data collection lasted from November to December 2020, reporting the time from October to December (representing the second wave), and the third data collection lasted from November to December 2021, evaluating the experience during the third wave from October to December 2021.

### 2.5. Statistical Analysis

The statistical analysis was conducted using SPSS statistical program version 26.0 (IBM Corp. Released 2019. Armonk, NY, USA). Two-way factorial ANCOVA was used to determine significance of interaction effect and main effects of pandemic period and the child’s diagnosis. T-test of two independent samples was used to determine specific differences between ASD and control group. Chi-square provided results of parental stressors and therapy availability during lockdown.

## 3. Results

### 3.1. Differences between Families with Neurotypical Children and Children with ASD during the Third Wave

During the third wave, parents of children with ASD scored higher in depression when compared to the parents of neurotypical children (t = 3.56, *p* < 0.001, d = 0.54). By contrast, the differences in stress (t = 1.78, *p* = 0.077, d = 0.27) and anxiety (t = 1.46, *p* = 0.146, d = 0.22) did not prove to be statistically significant. A more demanding child was rated the most prevalent parental stressor and was a problem for 74.1% of parents of children with ASD and 59.1% of parents of neurotypical children. Insufficient or unclear information was a prominent stressor for 63% of parents of children with ASD and 54.8% of parents of neurotypical children. The third most prevalent stressor was fears of the infection, more frequently in parents of neurotypical children (58.1%) than in the parents of children with ASD (50.6%). Less significant stressors include lockdown duration, frustration and boredom, unavailability of goods and services and work insecurity/finances, which were problematic for only 17–41% of respondents. Both internalizing (t = 6.84, *p* < 0.001, d = 1.03) and externalizing (t = 5.05, *p* < 0.001, d = 0.77) maladaptive behavior of children with ASD remained significantly higher when compared to neurotypical children.

### 3.2. Development from the First to the Third Pandemic Wave

#### 3.2.1. Depression, Anxiety and Stress of Parents

To analyze the changes in prevalence of depression, anxiety and stress in parents of children with ASD and parents of neurotypical children, we performed a two-way factorial analysis of covariance, as seen in the Table 2. We found the interaction effect of the pandemic period and the child’s diagnosis with stress (F (2,500) = 4.646, *p* = 0.010, η^2^ = 0.018) and depression (F (2,500) = 4.233, *p* = 0.015, η^2^ = 0.017). Interaction effect of the pandemic period with the diagnosis (ASD vs. neurotypical) was not observed for anxiety (F (2,500) = 2.222, *p* = 0.109, η^2^ = 0.009). In all cases we also recorded statistically significant main effects. A more detailed analysis showed the scores in all three subscales deteriorated in parents of children with ASD between the first and the second wave (D: t = 4.393, *p* < 0.001, d = 0.70; A: t = 3.648, *p* < 0.001, d = 0.58; S: t = 4.373, *p* < 0.001, d = 0.70), while the decline was confirmed exclusively in anxiety (t = 2.26, *p* = 0.025, d = 0.33) for parents of neurotypical children. During the second wave, we also observed statistically significant differences between the parents of children with ASD and of neurotypical children, in all three subscales (D: t = 4.70, *p* < 0.001, d = 0.75; A: t = 3.57, *p* < 0.001, d = 0.68; S: t = 4.26, *p* < 0.001, d = 0.57). There were no significant differences in stress, anxiety or depression of parents of children with ASD between the second and third wave. On the contrary, we observed a significant increase of stress in parents of neurotypical children (t = 2018, *p* = 0.045, d = 0.31). Therefore, during the third wave, both groups of parents only differed in the prevalence of depression, which remained higher in parents of children with ASD (t = (177) = 3.560, *p* < 0.001, d = 0.54).

#### 3.2.2. Parental Stressors

In general, “more demanding child care” was the most prevalent pandemic stressor for both groups of parents with maximum values of up to 78.87% for parents of ASD children and up to 59.14% for parents of neurotypical children. During the second wave, the importance decreased to 57.75% for parents of children with ASD and 37.80% for parents of neurotypical children and was replaced by “insufficient or unclear information” as the most prevalent stressor for both groups, selected by 42.25% of parents of children with ASD and 54.88% of parents of neurotypical children. The prevalence of “fears of the infection”, “lockdown duration” and “frustration and boredom” did not change significantly in time. “Work insecurity/finances” has proven to be a less significant stressor and its importance for the parents of neurotypical children declined over time from 34.15 to 27.96%, but grew from 35.21 to 40.74% for parents of children with ASD. “Unavailability of goods and services” has proven to be the least significant stressor, only chosen by 7.32–22.22% of participants. Complete information is included in Table 3.

#### 3.2.3. Maladaptive Behavior of Children

To analyze the changes in maladaptive behavior of children with ASD and neurotypical children, we performed a two-way factorial analysis of covariance. We did not observe the interaction effect of the pandemic period and the child’s diagnosis with internalizing (F (3,677) = 1.151, *p* = 0.328, η^2^ = 0.005) or externalizing maladaptive behavior (F (3,677) = 2.132, *p* = 0.095, η^2^ = 0.009). More detailed analysis of the main effects showed a significant effect of the diagnosis. Children with ASD scored significantly higher values in internalizing (F (3,677) = 203.022, *p* < 0.001, η^2^ = 0.231) as well as externalizing maladaptive behavior (F (3,677) = 101.215, *p* < 0.001, η^2^ = 0.130) during all data collections. The main effect of pandemic waves was lower and there were no statistically significant changes between the waves. Between the first and second wave, we found a significant increase in internalizing maladaptive behavior of children with ASD (t = 2.324, *p* = 0.021, d = 0.38). Contrastingly, in the group of neurotypical children, we recorded a statistically significant decrease of externalizing maladaptive behavior from the first to the second wave (t = 2.594, *p* = 0.010, d = 0.39). During the third wave, there were no significant changes for children with ASD, but for neurotypical children we found another increase almost to the original maximum from the first wave (t = 3.055, *p* = 0.003, d = 0.47), as stated in Table 4.

#### 3.2.4. Interventions

**Figure 1:**
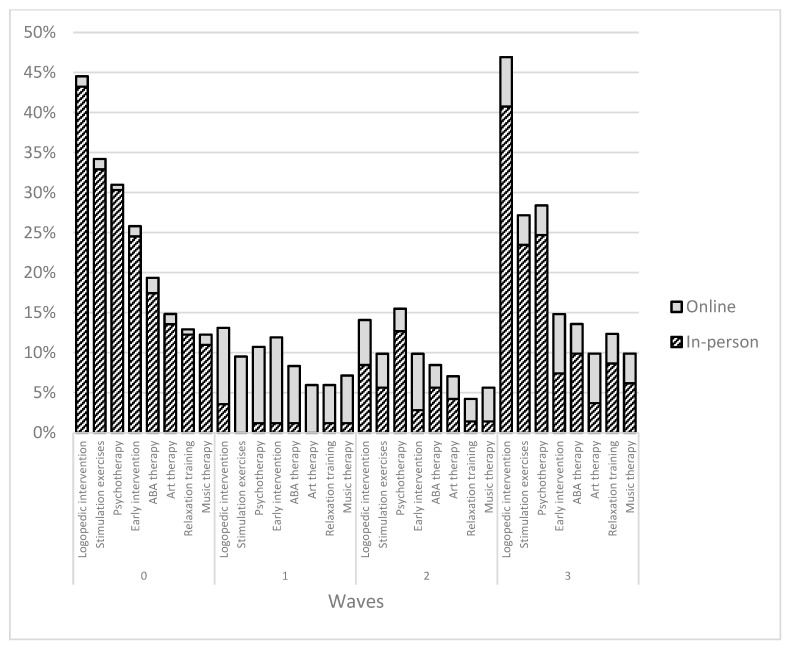
Percentage changes in attendance of various interventions for children with ASD, from pre-pandemic until the third wave.

Since the therapy availability restrictions caused by the pandemic were one of the biggest challenges for individuals with ASD, we also analyzed how the pandemic affected the availability of different interventions in Slovakia, as illustrated in Figure 1. Before the pandemic, logopedic therapy, stimulation exercises and psychotherapy were the most represented interventions. The first wave caused an extreme reduction of in-person interventions (max 13%) with only partial transition to the online form. During the second wave, there was a minor increase of in-person interventions such as logopedic therapy or psychotherapy, with a small increase in online interventions, too. During the third wave, we observed a massive increase of in-person therapies without any decrease in the ones offered online. Before the pandemic, 75.5% of children with ASD in our sample attended at least one type of intervention; this number dropped to 26.20% during the first wave and 31.0% during the second wave, but grew almost back to the pre-COVID numbers of 67.90% during the third wave.

## 4. Discussion

Even though numerous studies about the impact of the COVID-19 pandemic on mental health have been published in the past two years, it is not necessarily easy to generalize the information and extract what would help mitigate the negative influence in case of future pandemics. Research studies providing data about the experience of vulnerable populations and different countries with their particularities or using a longitudinal approach have proven to be the most valuable. Our research describes how the mental health of parents and children in Slovakia was affected during three waves of COVID-19 pandemic, primarily focusing on the unique experiences of children with autism spectrum disorder and their families. In line with other reports [5], our findings also indicate significant differences in the level of psychological distress experienced during each wave of the pandemic.

### 4.1. First Wave

While the first wave did not have a significant negative effect on the mental health of parents of neurotypical children, the parents of children with ASD already experienced high anxiety levels. More demanding child care was considered the strongest parental stressor, especially for parents of children with ASD. This is understandable in the context of the sudden closure of schools and facilities providing necessary interventions for the children, as well as a higher prevalence of both internalizing and externalizing maladaptive behavior of children with ASD when compared to neurotypical children, as reported in our findings and other studies [18,19,34,35]. Finding available therapies for children with ASD was challenging even before the pandemic, but due to the closure of services during the first wave we observed a significant decline in all forms of in-person interventions, with only a few available online.

### 4.2. Second Wave

During the second wave, both groups of parents experienced higher levels of anxiety, stress, and depression, but the prevalence was significantly higher in parents of autistic children. Deterioration in the mental health of adults was similarly reported in neighboring European countries [6,7,8,9,10] and in research focusing on parents of children with ASD [25,26]. Insufficient or unclear information was the most prevalent stressor for both groups of parents in our study, which is fitting to the way pandemic rules were being officially announced and how they changed rapidly and unexpectedly at the time. More demanding child care was the second most crucial stressor for parents of autistic children, while the lockdown duration took second place for parents of neurotypical children. Interestingly, the prevalence of externalizing maladaptive behavior did not change significantly between the first and second wave for children with ASD but decreased for children in the control group, who possibly learned to respect necessary restrictions better over time. On the contrary, internalizing maladaptive behavior grew significantly in autistic children. In means of interventions for children with ASD, we found a partial return to in-person form with some therapies provided online, but the availability remained generally low; only 31% of children attended at least one type of intervention.

### 4.3. Third Wave

There were no significant changes in the mental health of parents of children with ASD between the second and third wave, but we observed a significant increase in stress in parents of neurotypical children. Thus, during the third wave, the differences between both groups almost evened out, with no significant distinction in stress and anxiety but a persistently higher prevalence of depression in parents of children with ASD. More demanding child care was a growing stressor for both groups of parents during the third wave, followed by insufficient or unclear information and fears of the infection.

Similarly, we found an increase in externalized maladaptive behavior of neurotypical children and minimal changes in children with ASD. We can only hypothesize that it could be connected to neurotypical children becoming increasingly frustrated by the length of consecutive lockdowns. In contrast, autistic children may have already established new “pandemic routines” and were already adapted to a new standard of living through the pandemic. The improvement in the availability of therapies and the reopening of schools could be another possible explanation that prevented even further deterioration of the mental health in families with ASD children, something which occurred in the parents of neurotypical children. The otherwise expected increase in maladaptive behavior of children with ASD could be more easily managed by suitable interventions, which many of them finally received during the third wave.

As our results also show, the number of therapeutic interventions that ASD children received during the third wave was almost as high as before the pandemic, and we also found a massive increase for in-person therapies without the loss of online interventions. This is particularly important, because even though the situation is slowly getting better, the availability of specific interventions for children with ASD remains generally low in Slovakia. Therefore, especially for families living in smaller or distant cities, the online version of necessary interventions could be the solution even after the pandemic. Before the pandemic, 75.5% of children with ASD in our sample attended at least one form of intervention, and during the third wave this percentage grew back to 67.90%. The combined form of therapeutic intervention in-person and online proved to be an effective opportunity to continue services while adhering to social distancing guidelines [36]. During the pandemic, there were also first attempts to confirm the possibility of online autism assessment, either via parent-mediated video conferencing or via assessing videos of child’s behaviors [37]. In the future, this might be viable, especially for individuals with apparent autism traits or adults with ASD [38].

To sum up, we believe that in future research pandemic waves should be carefully considered when generalizing the information, as there seemed to be significant differences in the psychological distress experienced by individuals during each wave of the pandemic, presumably connected to various changes in safety measures and other variables. Our results clearly show that the COVID-19 pandemic had a negative impact on the mental health of many families in Slovakia, but the effect on families with children with autism spectrum disorder was more severe. Moreover, the changes in mental health development and coping with the COVID-19 pandemic differed for families with neurotypical children and families with children with ASD. The results of our research must be interpreted with regard to its limits, especially that it was a series of three cross-sectional studies as opposed to a longitudinal research, and data were collected online via self-report methods. This also led to the predominance of females in our sample, which could have affected our results, since in other studies [5,6,8] women reported more severe mental health issues during the COVID-19 pandemic. On the other hand, following the development of a situation in the same country over a two years period and three pandemic waves while focusing on the same particular research population is the greatest strength of this study.

## 5. Conclusions

Our findings suggest significant differences in the level of psychological distress experienced by people during the first, second and third waves of the COVID-19 pandemic in Slovakia. Furthermore, we also found that the mental health of families with neurotypical children and those with children with ASD progressed differently, plausibly because of how different pandemic situation rules affected their experience. This message should be considered while creating more sensible safety measures in the case of future pandemics.

## Figures and Tables

**Table 1:** Demographic characteristics of our sample recruited during the third COVID-19 pandemic wave and in total.

Wave	The Third Wave				Overall			
Parents								
Group	ASD		Control		ASD		Control	
Age Mean (SD)	39.51(7.23)		37.31(6.02)		39.4(6.96)		38.87(6.69)	
Males (*n*)	2		5		8		27	
Females (*n*)	79		88		228		243	
Children								
Group	ASD		Control		ASD		Control	
Count Total	81		93		236		270	
Child Gender	Boys	Girls	Boys	Girls	Boys	Girls	Boys	Girls
Count (*n*)	56	25	47	46	178	58	144	126
Age Mean (SD)	8.91 (3.78)	8.60 (4.29)	7.89 (3.68)	7.85 (4.44)	8.37 (3.72)	9.06 (4.30)	8.55 (4.30)	8.66 (4.50)

**Table 2:** Differences in depression, anxiety and stress between the parents of children with ASD and parents of neurotypical children.

	ASD	Control	t	*p*	d
First wave					
Depression	7.98 (9.20)	6.07 (8.30)	1.45	0.148	0.22
Anxiety	5.00 (7.14)	2.80 (3.80)	2.61	0.010	0.38
Stress	10.87 (8.95)	10.09 (7.97)	0.61	0.541	0.09
Second wave					
Depression	15.69 (12.61)	7.43 (9.03)	4.70	<0.001	0.75
Anxiety	10.08 (10.14)	4.87 (7.91)	3.57	<0.001	0.57
Stress	17.94 (11.19)	11.10 (8.66)	4.26	<0.001	0.68
Third wave					
Depression	14.84 (11.82)	9.13 (9.28)	3.56	<0.001	0.54
Stress	16.31 (9.78)	13.78 (8.90)	1.78	0.077	0.27
Anxiety	8.96 (8.89)	7.08 (8.15)	1.46	0.146	0.22

**Table 3:** Changes in the most prevalent stressors for the parents of children with ASD and parents of neurotypical children.

		The First Wave		The Second Wave		The Third Wave	
		ASD	Control	ASD	Control	ASD	Control
Lockdown duration	%	47.89	42.68	43.66	50.00	41.98	34.41
	(95% CI)	(35.80–60.08)	(31.82–54.10)	(31.91–55.95)	(38.75–61.25)	(30.99–52.96)	(24.57–44.25)
Fears of the infection	%	47.89	45.12	42.25	29.27	50.62	58.06
	(95% CI)	(35.80–60.08)	(34.10–56.51)	(30.61–54.56)	(19.74–40.35)	(39.49–61.74)	(47.85–68.28)
Frustration and boredom	%	42.25	40.24	30.99	28.05	37.04	36.56
	(95% CI)	(30.61–54.56)	(29.56–51.66)	(20.54–43.08)	(18.68–39.06)	(26.29–47.78)	(26.59–46.53)
Unavailability of goods and services	%	19.72	19.51	19.72	7.32	22.22	17.20
	(95% CI)	(11.22–30.86)	(11.58–29.74)	(11.22–30.86)	(2.73–15.25)	(12.97–31.47)	(9.39–25.02)
Insufficient or unclear information	%	42.25	54.88	61.97	74.39	62.96	54.84
	(95% CI)	(30.61–54.56)	(43.49–65.90)	(49.67–73.24)	(63.55–83.40)	(52.22–73.71)	(44.53–65.14)
Work insecurity/Finances	%	35.21	34.15	36.62	29.27	40.74	27.96
	(95% CI)	(24.24–47.46)	24.03–45.45)	(25.50–48.90)	(19.74–40.35)	(29.81–51.67)	(18.66–37.25)
More demanding child care	%	78.87	52.44	57.75	37.80	74.07	59.14
	(95% CI)	(67.56–87.56)	(41.11–63.59)	(45.44–69.39)	(27.32–49.19)	(64.32–83.82)	(48.96–69.32)

Note: CI = Confidence Interval.

**Table 4:** Differences in the internalizing and externalizing maladaptive behavior of children with ASD and neurotypical children.

	ASD	Control	t	*p*	d
First wave					
Maladaptive Internalizing behavior	9.40 (5.73)	4.56 (4.73)	6.19	<0.001	0.92
Maladaptive Externalizing behavior	5.75 (3.38)	3.66 (3.26)	4.20	<0.001	0.63
Second wave					
Maladaptive Internalizing behavior	11.54 (5.63)	4.71 (4.00)	8.74	<0.001	1.40
Maladaptive Externalizing behavior	6.20 (3.72)	2.48 (2.75)	7.09	<0.001	1.14
Third wave					
Maladaptive Internalizing behavior	11.00 (6.15)	5.46 (4.49)	6.84	<0.001	1.03
Maladaptive Externalizing behavior	6.89 (3.83)	4.00 (3.71)	5.05	<0.001	0.77

## Data Availability

The data collected in this study can be obtained from the corresponding author upon reasonable request.
